# Molecular pathogenesis, global epidemiological trends, and treatment strategies for pteropine orthoreoviruses: a narrative review

**DOI:** 10.1080/22221751.2026.2732705

**Published:** 2026-09-10

**Authors:** Francesco Branda, Giancarlo Ceccarelli, Ranjan K. Mohapatra, Mohamed Mustaf Ahmed, Uthman Okikiola Adebayo, Dong Keon Yon, Antonello Maruotti, Fabio Scarpa, Massimo Ciccozzi

**Affiliations:** a Unit of Medical Statistics and Molecular Epidemiology, Università Campus Bio-Medico di Roma, Rome, Italy; b Department of Public Health and Infectious Diseases, University Hospital Policlinico Umberto I, Sapienza University of Rome, Rome, Italy; c Department of Chemistry, Government College of Engineering, Keonjhar, Odisha, India; d Faculty of Medicine and Health Sciences, SIMAD University, Mogadishu, Somalia; e Department of Medical Laboratory Services, Federal University of Health Sciences Teaching Hospital, Ila-Orangun, Nigeria; f Department of Digital Health, Global Health Focus Africa, Kigali, Rwanda; g Center for Digital Health, Medical Science Research Institute, Kyung Hee University College of Medicine, Seoul, South Korea; h Department of Precision Medicine, Kyung Hee University College of Medicine, Seoul, South Korea; i Department of Pediatrics, Kyung Hee University College of Medicine, Seoul, South Korea; j Department of Public Health and Epidemiology, Khalifa University, Abu Dhabi, UAE; k Department GEPLI, LUMSA University, Rome, Italy; l Center for Biotechnology, Khalifa University, Abu Dhabi, UAE; m Department of Biomedical Sciences, University of Sassari, Sassari, Italy

**Keywords:** Pteropine orthoreovirus, bat-borne viruses, zoonotic spillover, fusion-associated small transmembrane proteins, molecular pathogenesis, emerging infectious diseases, public global health, viral reassortment

## Abstract

Pteropine orthoreoviruses are emerging bat-borne zoonotic viruses of the genus *Orthoreovirus* (family *Reoviridae*), increasingly recognized as causes of acute respiratory disease in humans. Originally grouped with the largely non-pathogenic mammalian orthoreoviruses, they have challenged that view through their association with severe influenza-like illness, evidence of human-to-human transmission, and a broad geographic range across the Old World. Maintained primarily in fruit bats of the family *Pteropodidae*, they are now linked to neurological as well as respiratory disease. This narrative review synthesizes current knowledge of their molecular pathogenesis, zoonotic ecology, and global epidemiology, integrating recent advances in phylogeography, reassortment-driven evolution, spillover dynamics, and translational biomedical applications within a unified One Health framework. Genomic diversity, reassortment potential, and the unique fusion-associated small transmembrane proteins together underpin viral adaptability and pathogenicity. Major gaps nonetheless remain in transmission dynamics, host adaptation, shedding ecology, and pandemic potential. Future priorities should include integrated genomic surveillance, improved diagnostic strategies, validated experimental models, and interdisciplinary One Health approaches to strengthen outbreak preparedness and prevention.

## Introduction

Zoonotic viruses from wildlife reservoirs remain a persistent public-health threat [1]. Bats are particularly important reservoirs because they host diverse viruses and often tolerate infection, likely due to ecological traits and immune adaptations that limit immunopathology [2–4]. Within bat-borne viruses, *pteropine orthoreoviruses* (PRVs) are increasingly relevant, yet still under-recognized, causes of human respiratory disease [5]. Orthoreoviruses (family *Reoviridae*) are non-enveloped viruses with segmented dsRNA genomes enclosed in multilayered icosahedral capsids [6]. Historically, mammalian reoviruses were viewed as largely benign “orphan” viruses, contributing to limited routine testing and surveillance [7]. This view shifted with the discovery of fusogenic, fruit-bat-associated orthoreoviruses: Nelson Bay virus was isolated in Australia in 1968 [8], and related PRVs were later detected across Asia and Africa, including isolation from patients with acute respiratory illness and occasional limited human-to-human transmission [9,10]. PRVs differ from classical mammalian reoviruses (MRVs) by encoding fusion-associated small transmembrane (FAST) proteins that drive syncytium formation, promoting rapid cell-to-cell spread and cytopathic effects; FAST-deficient viruses are attenuated, underscoring their role in pathogenesis [11–13]. PRV evolution is also facilitated by a 10-segment genome that supports reassortment and diversification in bat reservoirs [14,15]. Mechanistically, broad host range may be aided by attachment to ubiquitous heparan sulphate proteoglycans [16].

From both clinical and public health perspectives, PRV infections are difficult to identify because their symptoms often resemble those of common respiratory illnesses. In addition, orthoreoviruses are rarely included in routine diagnostic testing, which likely leads to underdiagnosis and limits accurate assessment of transmission dynamics across different settings and viral strains [17]. At the same time, the combination of reservoir persistence, reassortment-driven diversity, and broad attachment factors supports ongoing opportunities for spillover and sporadic clusters, making PRVs relevant to surveillance strategies for severe acute respiratory infection and encephalitis of unknown etiology, especially in regions where bat–human contact is frequent [18,19].

Despite growing recognition of PRVs as emerging zoonotic pathogens, the current literature remains fragmented, with most studies focusing separately on virology, isolated outbreak reports, or reservoir ecology. Few reviews have comprehensively integrated the molecular mechanisms of pathogenicity with global phylogeographic expansion, zoonotic transmission dynamics, and translational biomedical applications [17,20]. Moreover, recent evidence on African PRV circulation, reassortment-associated evolution, neurological manifestations, and advances in reverse genetics have not been synthesized within a unified framework [21]. This review addresses these gaps by providing an integrative evaluation of PRVs through a One Health perspective linking viral evolution, ecological disruption, surveillance challenges, and emerging therapeutic opportunities. Accordingly, this review aims to present the molecular pathogenesis, zoonotic dynamics, and global epidemiological trends of PRVs while highlighting key surveillance gaps and public health implications.

## Taxonomic classification, genomic architecture, and pathogenesis of the *pteropine orthoreovirus* species

### Taxonomic classification and genomic organization of PRV

**Figure 1.**
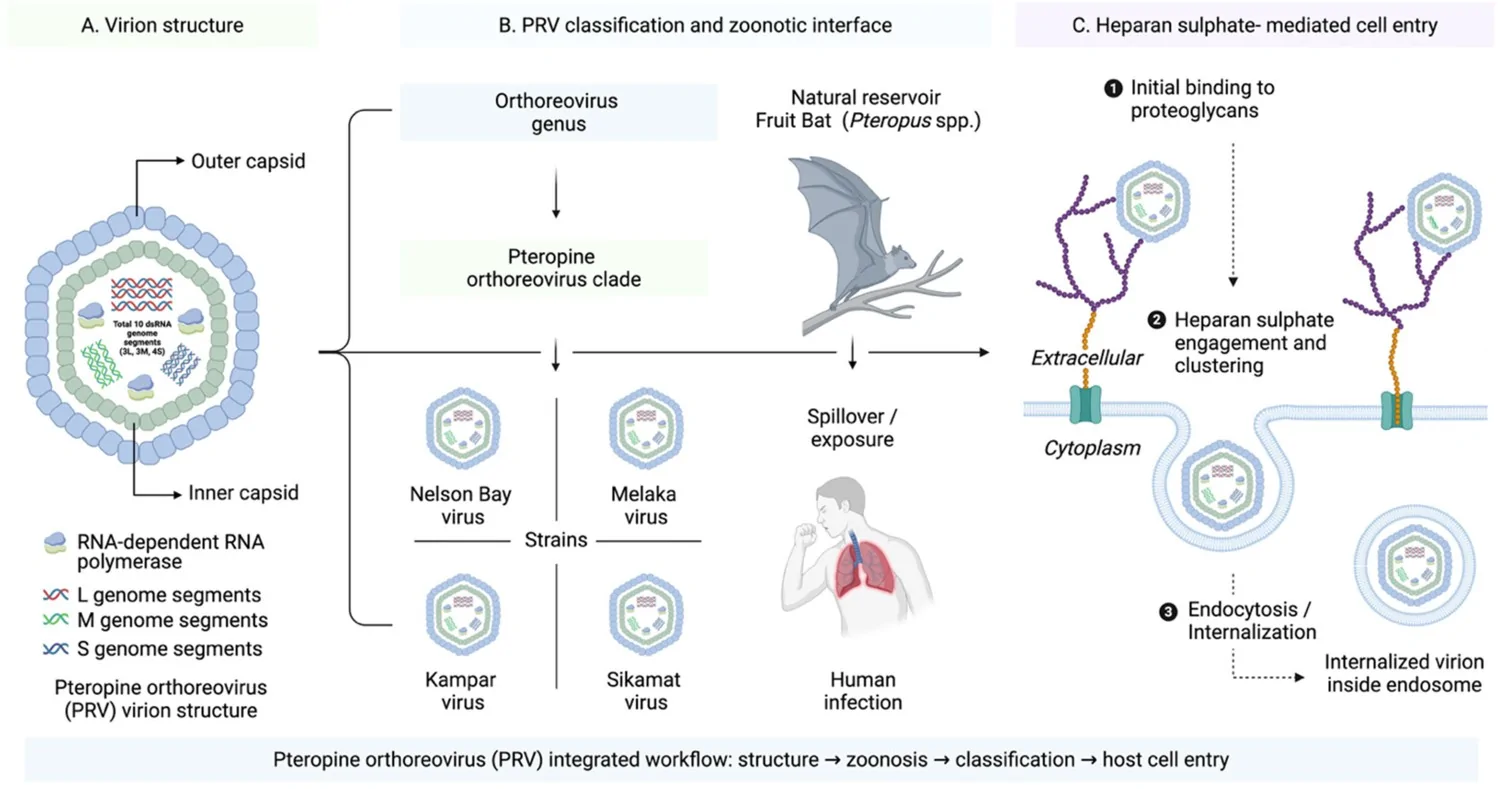
Structure, classification, and host cell entry mechanism of *Pteropine orthoreovirus (*PRV). (A) Virion architecture: The PRV virion is a non-enveloped, double-layered icosahedral particle (60–80 nm in diameter). The outer capsid (composed of proteins such as *σ*B and *σ*C) mediates receptor binding and entry, while the inner capsid (core) encloses the ten segments of double-stranded RNA genomic material (categorized as Large/L, Medium/M, and Small/S segments) along with the viral RNA-dependent RNA polymerase. (B) Representative strains and zoonotic context: Key PRV strains (e.g. prototype Nelson Bay virus, human-pathogenic Melaka and Kampar viruses) are depicted in relation to their natural reservoir (fruit bats of the genus *Pteropus*) and the spillover event leading to human infection. (C) Heparan sulphate-mediated entry pathway: Viral attachment is initiated by the electrostatic interaction between the outer capsid proteins and negatively charged heparan sulphate proteoglycans on the host cell surface. Following heparan sulphate engagement and clustering, the virion is internalized via clathrin-mediated endocytosis, resulting in an internalized virion within an endosomal compartment, the first critical step of infection. Created in BioRender. Ahmed, M. M. (2026) https://BioRender.com/jep09ay.

The genus *Orthoreovirus*, within the subfamily *Spinareovirinae* of the family *Reoviridae*, encompasses non-enveloped viruses characterized by a segmented dsRNA genome enclosed in a double-layered icosahedral protein capsid [22]. Taxonomically, the genus is primarily divided based on the phenotypic ability to induce cell–cell fusion, creating two distinct groups: the non-fusogenic MRV and the fusogenic orthoreoviruses, which include the avian, baboon, and reptilian orthoreoviruses and those isolated from bats, as summarized in Table 1 and schematically represented in Figure 1.

**Table 1.** Taxonomic hierarchy and defining characteristics of *Pteropine orthoreovirus (PRVs)* within the family *Reoviridae*. PRV strain nomenclature generally reflects the order of identification together with an abbreviation linked to the isolate name or geographic origin. For example, PRV1N corresponds to the prototype Nelson Bay virus isolate, PRV2P to Pulau virus, PRV3M to Melaka virus, PRV4 K to Kampar virus, PRV7S to Sikamat virus, and PRV16 K to the Ugandan strain identified in Kasokero cave bat populations.

Taxonomic hierarchy	Classification	Significant feature
Family	*Reoviridae*	Double-layered icosahedral capsid, 10–12 dsRNA segments
Subfamily	*Spinareovirinae*	Presence of large turrets at the icosahedral vertices
Genus	*Orthoreovirus*	10 dsRNA segments, vertebrate hosts only
Species	*Pteropine orthoreovirus*	Fusogenic, primarily bat-borne, zoonotic potential
Representative strains	PRV1N, PRV2P, PRV3M, PRV4 K, PRV7S, PRV16K	Named by isolate order and location of discovery

The species PRV was formally proposed to the International Committee on Taxonomy of Viruses to unify several closely related isolates recovered from bats and humans, replacing older designations such as Nelson Bay virus (NBV), which now serves as the prototype isolate for the PRV group [23]. The reclassification was necessitated by high genetic similarity, overlapping host ranges among pteropid bats, and strong serological cross-reactivity between isolates such as Melaka, Kampar, Pulau, and the original NBV strain [23]. Phylogenetic analyses of both outer and inner capsid proteins consistently classify PRVs within a distinct lineage (group III) that is clearly separate from classical non-fusogenic mammalian orthoreoviruses. Among the genome segments, the greatest genetic diversity is observed in the S1 segment [23,24]. Despite this marked variability, conserved terminal sequences are retained, including the characteristic 5’-end consensus motif 5’-GCUUUU(U/C) and the 3’-end sequence 5’-UCAUC-3’, which remain defining molecular signatures of the genus [25].

The PRV genome consists of 10 linear dsRNA segments, conventionally divided into three size classes based on electrophoretic mobility: Large (L1, L2, L3), Medium (M1, M2, M3), and Small (S1, S2, S3, S4) [23]. These segments encode both the structural components of the virion and a set of non-structural proteins essential for viral replication, cell entry, and host-cell manipulation, as detailed in Table 2. The PRV virion is a non-enveloped particle, approximately 60–80 nm in diameter, displaying icosahedral symmetry and a characteristic double-layered capsid architecture [16]. The inner shell, or core, encloses the ten dsRNA genome segments together with the enzymatic machinery required for transcription, including the RNA-dependent RNA polymerase (RdRp), while the outer capsid shell mediates receptor binding, cell entry, and protection of the viral genome [22].

**Table 2.** Genomic organization of *Pteropine orthoreovirus* and functional annotation of proteins encoded by the ten double stranded RNA segments.

Segment class	Segment	Protein name	General functional category
Large (L)	L1, L2, L3	*λ*A, *λ*B, *λ*C	Core proteins; RdRp; turret protein (guanylyltransferase)
Medium (M)	M1, M2, M3	*µ*A, *µ*B, *µ*C	Capsid proteins; primary outer capsid components
Small (S)	S1	p10, p17, *σ*C	FAST protein (fusion); non-structural regulatory; cell attachment
	S2	*σ*A	Core/inner capsid scaffold protein
	S3	*σ*B	Outer capsid protein
	S4	*σ*NS	Non-structural; RNA-binding; replication facilitation

A defining characteristic of PRVs is the polycistronic organization of specific genome segments, most notably S1 and, in some isolates, S4 [23]. While the majority of orthoreovirus genome segments are monocistronic, the S1 segment of PRV, which is approximately 1568 bp in length, typically contains three distinct open reading frames encoding the p10 FAST protein, the non-structural p17 protein, and the *σC* cell attachment protein [13,23]. This compact and information-dense genomic arrangement enables PRVs to maximize coding capacity within a limited segmented genome and supports the coordinated expression of proteins involved in membrane fusion, host-specific regulation, and viral attachment, contributing to viral adaptability and zoonotic potential [26].

### Molecular mechanisms of infection and pathogenesis

PRVs initiate infection by attaching to cell-surface heparan sulphate proteoglycans (HSPGs), a strategy that differs from the junctional adhesion molecule 1 (JAM-1) receptor paradigm of mammalian orthoreoviruses [16]. Attachment is mediated by outer capsid proteins through electrostatic interactions with negatively charged heparan sulphate (HS) chains, followed by internalization (typically via clathrin-mediated endocytosis) [16]. Evidence for HS dependence comes from genetic and enzymatic perturbation of HS biosynthesis or structure, which markedly reduces viral attachment and infection [16]. Clinically, reliance on ubiquitous HSPGs may contribute to broad tissue tropism and suggests entry-blocking strategies (e.g. HS competitors) as a therapeutic avenue.

**Figure 2.**
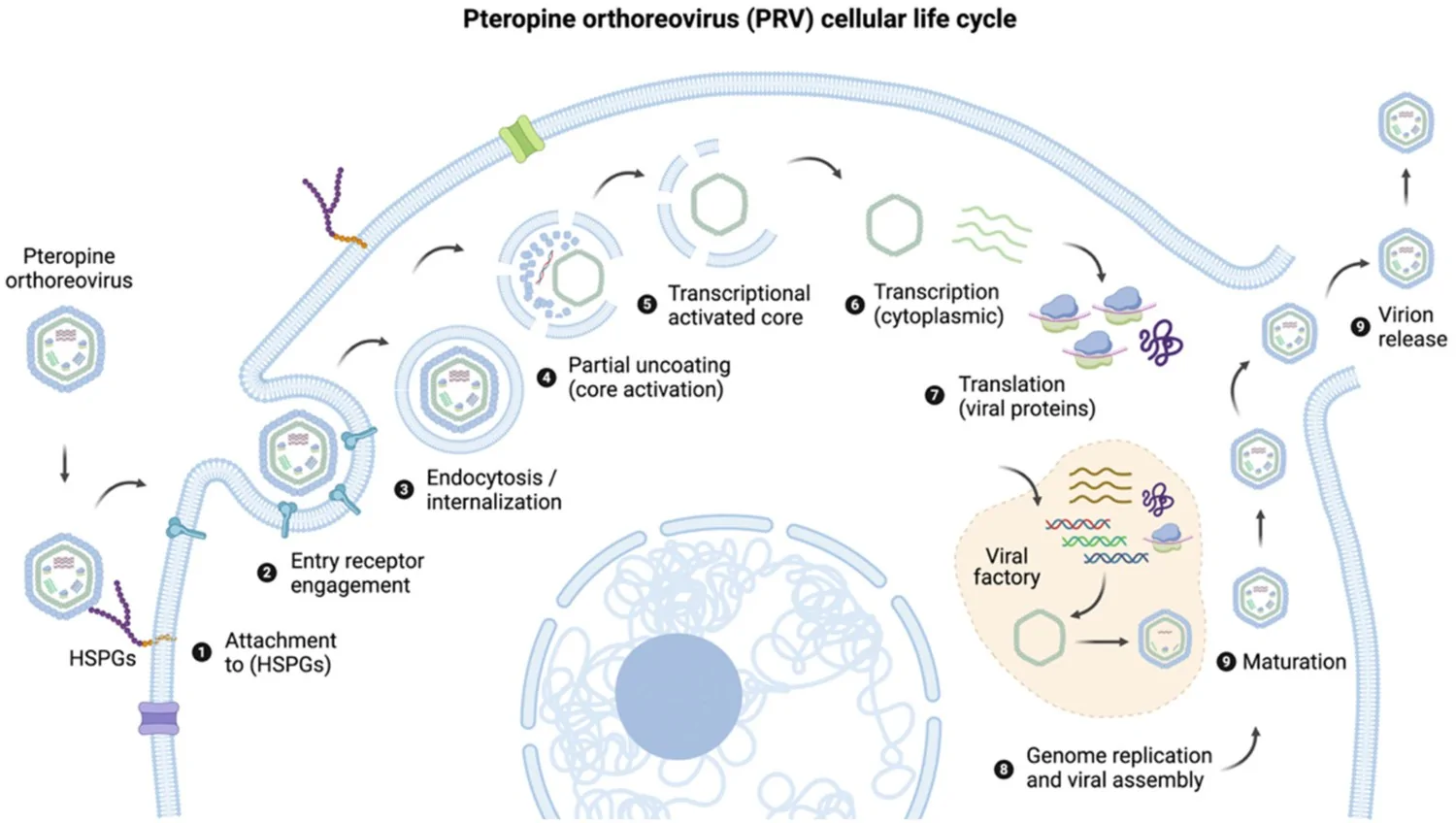
Schematic representation of the cellular life cycle of *Pteropine orthoreovirus* (PRV). The PRV life cycle begins with viral attachment to heparan sulphate proteoglycans on the host cell surface via outer capsid proteins, followed by clathrin-mediated endocytosis. After partial capsid disassembly within endosomes, the transcriptionally active core is released into the cytoplasm, where viral double stranded RNA is transcribed and translated. Newly synthesized genomic segments are assembled into progeny virions. A key pathogenic feature is the expression of the non-structural p10 protein, which induces cell-cell fusion and syncytium formation, facilitating direct viral spread and immune evasion. Mature virions are subsequently released to initiate new rounds of infection. Created in BioRender. Ahmed, M. M. (2026) https://BioRender.com/d0dagnu.

After entry, PRVs are distinguished by their ability to induce extensive cell–cell fusion and multinucleated syncytia, driven by the non-structural FAST protein p10 expressed at the plasma membrane of infected cells [27]. FAST-mediated fusion enables efficient local spread between adjacent cells and is supported by experimental evidence showing that disruption of FAST function reduces syncytium formation and attenuates both replication and pathogenicity [13]. Clinically, syncytiogenesis likely contributes to epithelial damage and respiratory pathology, and highlights interactions between the fusion machinery and host factors as potential intervention targets. The PRV life cycle, integrating HSPG-mediated entry and FAST-driven cell-to-cell spread, is summarized in Figure 2.

The p10 FAST protein itself is a small, non-structural type III membrane protein with a short, mostly hydrophobic ectodomain, a single transmembrane domain, and a cytoplasmic tail. Unlike enveloped-virus fusogens, it does not require a distinct receptor-binding step; instead, once it accumulates at the plasma membrane and at sites of cell–cell contact, it directly lowers the energy barrier for lipid bilayer merger between adjacent cells [11,12]. This mechanistic distinction underlies its dual role, noted above, in local viral spread and, as discussed in Section 4, in oncolytic and biotechnological applications that exploit FAST-mediated bystander cell killing as a potential therapeutic strategy.

Beyond entry and fusion, PRV host range and reservoir persistence is shaped by the non-structural p17 protein encoded on the S1 segment. p17 localizes to the nucleus and is dispensable for replication in standard cell lines but supports efficient growth in bat-derived cells; this phenotype depends on basic-residue motifs required for nuclear localization [26]. p17 can also modulate host responses: Micafungin-mediated suppression of p17 has been linked to upregulation of interleukin-6 (IL-6), which in turn can interfere with syncytium formation and viral release [28]. Together, these findings suggest p17 is a molecular determinant of host specificity and a contributor to the largely asymptomatic reservoir state in fruit bats.

A further determinant of virulence lies within the inner capsid. The protein σA, encoded by the S2 segment, has recently been identified as a major contributor to pathogenicity. Using reverse genetics to exchange genome segments between a highly virulent human-derived PRV strain and a low-virulence bat-derived strain, σA was shown to drive an excessive, dysregulated inflammatory response in infected mice, with pathological changes evident in lung tissue. A single conserved serine residue in σA (Ser-46) is present in PRV strains isolated from humans, monkeys, and bat flies, but is absent from bat-derived strains, suggesting that this residue may facilitate interspecies transmission from the natural bat reservoir to incidental hosts [29]. How often these entry, fusion, and virulence determinants are presented to human hosts is governed in turn by the ecology of the reservoir itself, which is considered next.

## Natural reservoir dynamics and global epidemiology of *pteropine orthoreoviruses*

### The *Pteropodidae* family as primary reservoir hosts

The natural reservoir dynamics of PRVs are intrinsically linked to the biology and ecology of bats within the family *Pteropodidae*, commonly known as Old World fruit bats or flying foxes [30]. These mammals not only persistently host the virus but also facilitate its genetic diversification through co-infection mechanisms and genomic reassortment [17]. Members of this family exhibit remarkable susceptibility to PRV infection, with evidence spanning numerous genera and species across continents (Table 3). In Australia, the primary reservoir remains *Pteropus poliocephalus*, while in Southeast Asia, host diversity expands to include *Pteropus vampyrus*, *Pteropus hypomelanus*, and *Rousettus leschenaultii* [16]. African studies have recently identified PRV in *Lissonycteris angolensis*, *Rousettus aegyptiacus*, and *Eidolon helvum*, suggesting viral endemicity across much of the continent’s fruit bat populations [21].

**Table 3.** Reservoir hosts and documented human events associated with Pteropine orthoreovirus.

Context	Year(s)	Location	Species / Isolate	Cases or Seroprevalence	Notes	Ref(s)
Reservoir	–	Australia	*Pteropus poliocephalus* / Nelson Bay virus (NBV)	Historical (initial isolation)	Prototype isolate for the PRV species	See Section 3.1
Reservoir	–	Malaysia (Tioman Island)	*Pteropus hypomelanus* / Pulau virus (PRV2P)	12.8–17.8% seroprevalence	–	See Section 3.1
Reservoir	–	Indonesia	*Pteropus vampyrus* / Indonesia/2010 isolate	Consistent detection in fecal samples	–	See Section 3.1
Reservoir	–	Philippines	*Eonycteris spelaea* / PRV-Samal-24	83% neutralizing antibodies	–	See Section 3.1
Reservoir	–	China	*Rousettus leschenaultii* / Xi River–Cangyuan virus	High genetic heterogeneity	–	See Section 3.1
Reservoir	–	Uganda	*Lissonycteris angolensis* / PRV16K	Recent discovery (metagenomics)	–	See Section 3.1
Reservoir	–	Zambia	*Rousettus aegyptiacus* / Nachunsulwe-57	98% seropositivity	–	See Section 3.1
Reservoir	–	Zambia / Ghana	*Eidolon helvum* / –	–	High diversity of paramyxoviruses and reoviruses	See Section 3.1
Human	2006	Malaysia (Melaka)	Melaka virus	2 (ILI, RTI)	First documented human spillover; bat-to-human (C), limited human-to-human (*P*)	[9]
Human	2006	Malaysia (Kampar)	Kampar virus	4 (ILI, RTI)	Human-to-human (C), nosocomial cluster	[33]
Human	2007–2010	Indonesia (Bali) / Japan / Hong Kong (imported)	Miyazaki-Bali and Hong Kong strains	2 (Acute RTI)	Bat exposure (P)	[34]
Human	2011	Malaysia (Sikamat)	Sikamat virus (PRV7S)	1 (RTI)	Bat exposure (P); later characterized as oncolytic candidate (Section 4)	[10,35]
Human	2016–2019	Malaysia (multisite)	Surveillance study (Tee et al.)	20+ (PCR+)	Environmental/bat contact (P)	[36]
Human	2019	Singapore / Malaysia	Serosurvey (Tan et al.)	15% (Singapore forestry workers); 5% (Malaysia)	Occupational exposure serosurvey; see methodological critique in Section 3.3	[37]
Human	2022–2023	Bangladesh (3 districts)	First PRV-associated fatal encephalitis cluster	At least 11 cases; CFR 36%	Acute respiratory illness + encephalitis; palm sap contamination (bat excreta) (P)	[38]

ILI = influenza-like illness; RTI = respiratory tract infection; CFR = case fatality rate; *P* = presumed; C = confirmed. For the Kampar cluster, the number of cases is reported as at least 4 in the literature [33]. Reservoir entries are referenced collectively to Section 3.1.

The tropism of PRVs within reservoir hosts is multicentric. Successful virus isolation has been achieved from various biological samples including cardiac blood, urine, lung tissues, intestinal contents, and saliva [24,31]. This systemic distribution suggests intra-colony transmission can occur through multiple routes, including respiratory (via droplets) and fecal-oral pathways, increasing the probability of viral persistence within high-density colonies [31]. Infection dynamics within bat populations are characterized by high antibody prevalence, often exceeding 90% in some African and Asian populations, indicating endemic circulation and frequent re-exposure [21]. Despite occasional high viral loads detected in samples, bats typically show no clinical signs of disease, acting as asymptomatic carriers that shed virus intermittently [32].

**Figure 3.**
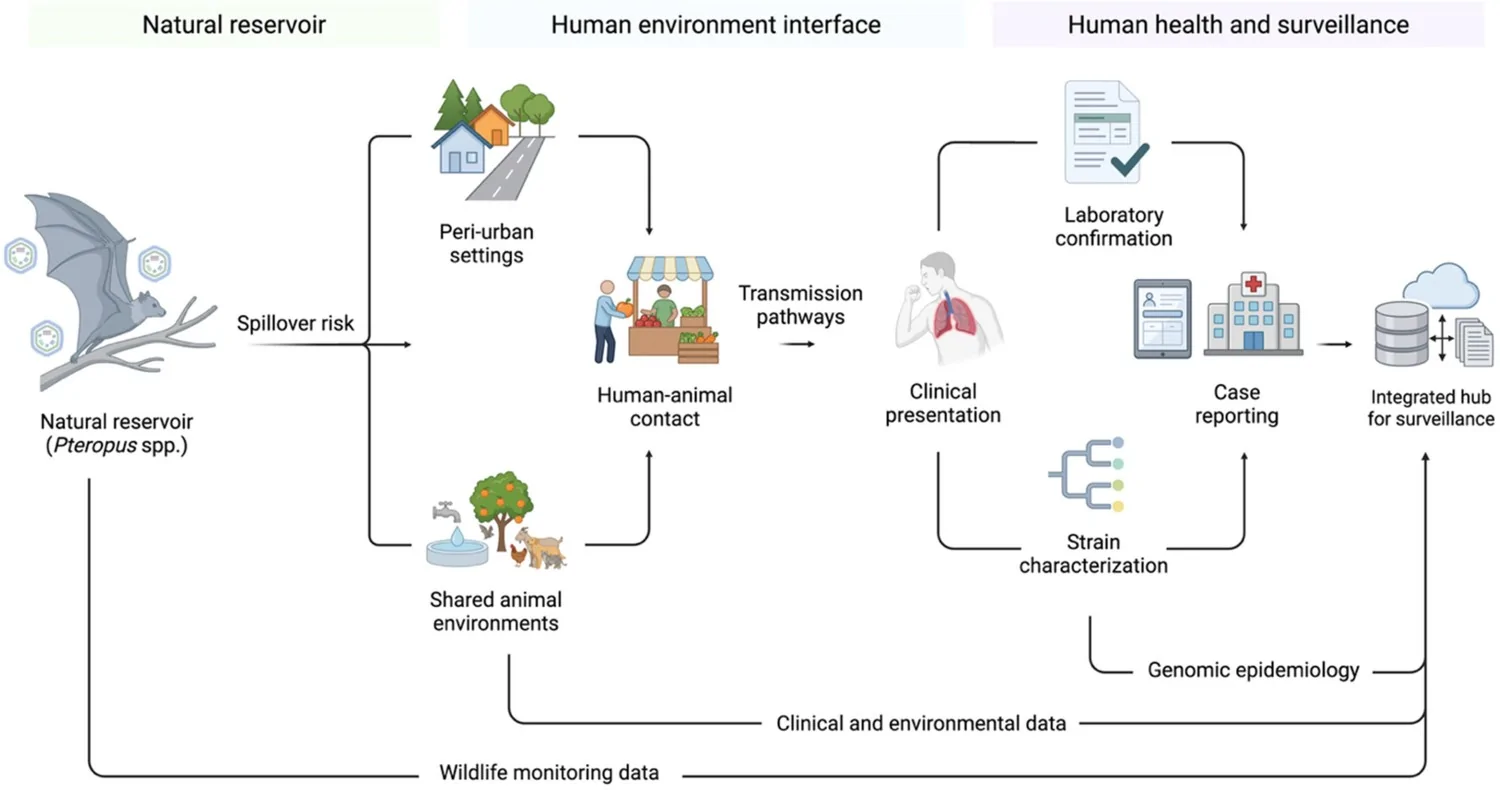
Zoonotic transmission pathways and integrated One Health surveillance framework for *Pteropine orthoreoviruses* (PRVs). The schematic illustrates the multi-host ecology, transmission pathways, and One Health surveillance framework of PRVs. Fruit bats of the family *Pteropodidae* serve as the primary reservoir hosts, maintaining asymptomatic viral circulation. Human spillover may occur through direct contact with bats or their excreta, environmental exposure to contaminated aerosols or fomites, consumption of contaminated food products, or via intermediate hosts such as macaques. Human infection can result in respiratory or neurological disease, with limited human-to-human transmission documented in some settings. The figure also highlights integrated surveillance and intervention strategies, including animal and environmental monitoring, clinical surveillance, laboratory diagnostics, public health education, habitat conservation, infection control measures, and rapid outbreak response. Laboratory confirmation and strain characterization are shown in outline only; the diagnostic assays and their clinical application are detailed in Figure 5. Created in BioRender. Ahmed, M. M. (2026) https://BioRender.com/pv2gro5.

A crucial element in reservoir dynamics is the social behaviour of pteropodid bats. Many species, such as *Pteropus vampyrus* and *Eidolon helvum*, form massive colonies numbering thousands or even millions of individuals, often in close physical proximity [2,39]. This crowding facilitates horizontal transmission through frequent contact and aerosolized secretions. Furthermore, the nomadic nature of many flying foxes, which travel vast distances following seasonal flowering patterns, promotes viral spread between distant colonies, maintaining a highly dynamic viral genetic pool [21]. Research from China has introduced additional complexity, with PRV strains isolated from bat flies (*Eucampsipoda sundaica*), hematophagous ectoparasites collected from bat surfaces [40]. While their role as biological vectors to humans remains unconfirmed, their ability to harbour virus suggests they may act as mechanical or biological vectors between bats themselves, facilitating virus maintenance during periods of low flight activity or hibernation [40]. The intricate network of zoonotic transmission routes and the essential framework for effective surveillance according to the One Health approach are summarized in Figure 3.

### Global geographic distribution, phylogeography, and epidemiological evolution of *pteropine orthoreoviruses*

The understanding of PRV geographic distribution has evolved from an initially regionalized perspective, largely confined to Australia and Southeast Asia, to a global recognition of viral presence across nearly all regions inhabited by pteropodid bats. Australia represents the historical origin of PRV research, with the isolation of NBV in New South Wales in 1968 [9]. A major epidemiological shift became evident in Southeast Asia, which subsequently emerged as the principal epicentre of human PRV infections. Malaysia has yielded a particularly rich collection of isolates, including Pulau virus (1999), Melaka virus (2006), Kampar virus (2006), and Sikamat virus (2011) [10]. These viruses were isolated not only from bats but also from patients with severe acute respiratory infections, conclusively demonstrating the zoonotic nature of PRVs. PRV circulation has been documented across multiple regions through both direct isolation from bats and travel-associated human infections, indicating sustained viral circulation and ongoing zoonotic exposure [34]. Surveillance studies have identified genetically diverse PRV strains in bat populations, suggesting the emergence of distinct regional lineages potentially shaped by geographic isolation and reassortment events [17,24]. Serological evidence further indicates that human exposure to PRVs may be more common than currently recognized and is often asymptomatic or clinically unrecognized [37]. In addition to fruit bats, PRVs have also been detected in other animal hosts, highlighting their ecological adaptability and potential for broader transmission dynamics [41]. More recently, the identification of PRV strains in Africa substantially expanded the recognized geographic range of these viruses beyond their previously assumed distribution [17]. Despite their geographic separation, African and Asian PRV strains share striking genetic similarity, often exceeding 91% nucleotide identity across multiple genome segments, supporting the existence of a pan–Old World continuum of PRV diversity [17].

Global phylogeographic analyses further reinforce this continuum model. A significant correlation between geographic distance and genetic divergence, consistent with isolation by distance, has been demonstrated through Mantel tests applied to genomic segments S1–S4, showing that genetic distance increases linearly with geographic separation even at intercontinental scales of up to 19,420 km [17]. However, evolutionary rates differ among genome segments. The S1 segment, encoding the cell attachment protein σC, exhibits the greatest divergence, with an average pairwise distance of 35% ± 12.7%, likely reflecting diversifying selection driven by adaptation to host-specific cellular receptors [17]. In contrast, the S2 and S4 segments are more conserved, suggesting stronger structural and functional constraints on inner and outer capsid proteins [17]. The segmented genome organization of PRVs creates the potential for genomic reassortment, which may contribute to viral diversification and geographic spread. Several studies have reported phylogenetic incongruence among individual genome segments of PRV isolates, suggesting possible reassortment events during viral evolution [17,19,36,42]. For example, strains exhibit varying phylogenetic relationships depending on the genome segment analyzed, indicating complex evolutionary dynamics and possible historical exchange of genomic segments among circulating lineages [36,43]. However, comprehensive genomic investigations remain limited, and the extent to which reassortment directly influences PRV emergence, host adaptation, and pathogenicity requires further study [44].

Parallel to this expanding geographic and genetic landscape, the epidemiology of PRVs has evolved substantially. Following the initial discovery of NBV in 1968, a pivotal milestone occurred in 2006 with the first documented human spillover event associated with Melaka virus, confirming the zoonotic potential of PRVs [9]. In the same year, the Kampar cluster provided evidence of limited human-to-human and nosocomial transmission, demonstrating that PRVs are capable not only of animal-to-human spillover but also of secondary transmission under specific conditions [33]. Between 2007 and 2010, imported cases linked to strains such as Miyazaki-Bali and Hong Kong isolates further confirmed endemic circulation in Indonesia and highlighted the role of international travel in disseminating infections beyond their region of origin [45]. More recently, reports from Bangladesh described clusters of severe respiratory and neurological illness potentially associated with PRV exposure, further expanding the recognized clinical and geographic spectrum of these viruses [46,47]. These findings raised concerns regarding the possible involvement of PRVs in encephalitic disease and highlighted the need for enhanced surveillance and confirmatory molecular investigations.

**Figure 4.**
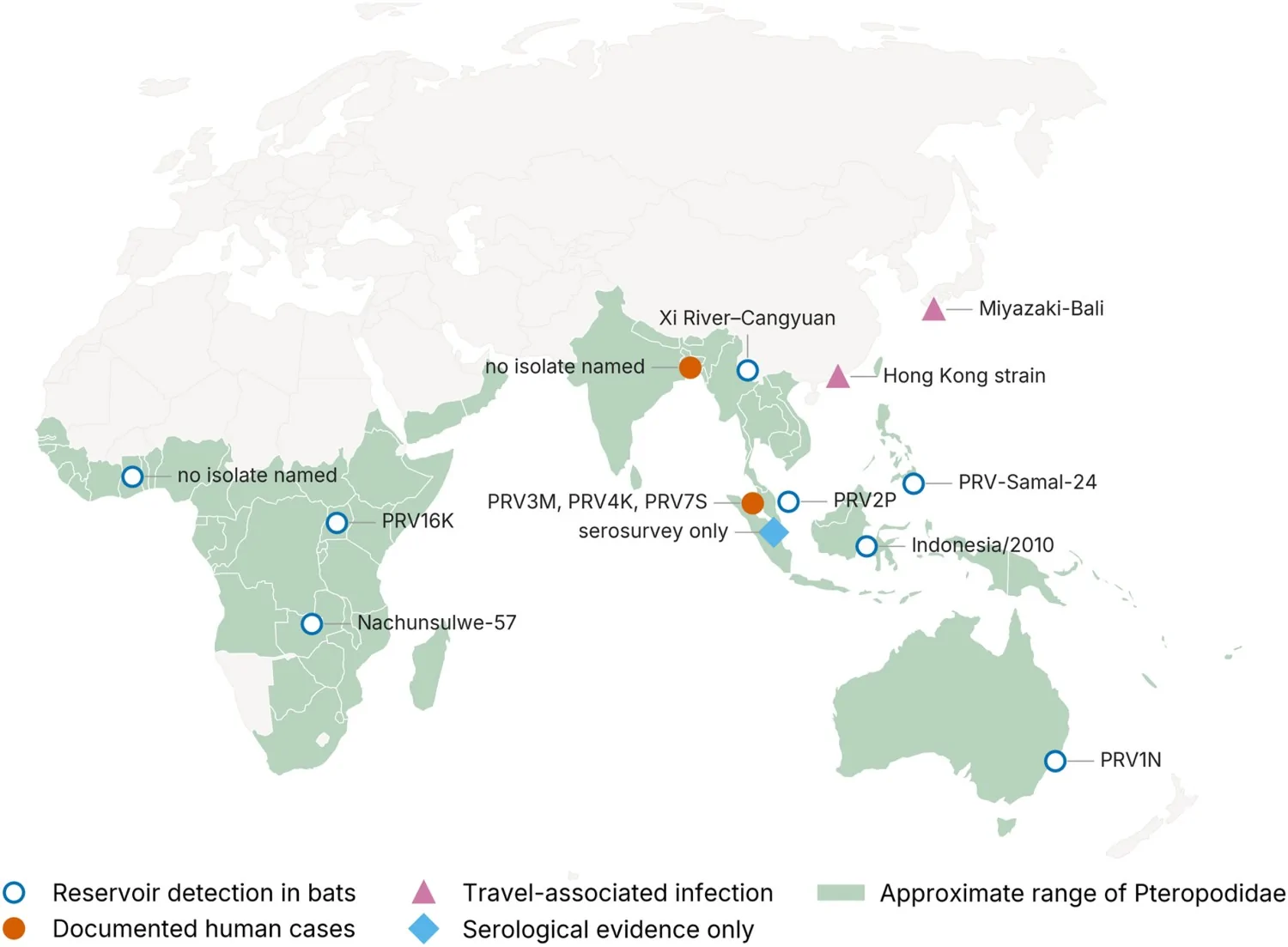
Global distribution of Pteropine orthoreovirus (PRV) strains. Detections are shown on a Robinson projection of the Old World, against the approximate range of the pteropodid bat reservoir (shaded). Marker shape and colour indicate the class of evidence, and each marker is annotated with the strain reported at that site; Ghana and Bangladesh are annotated “no isolate named”, and Singapore “serosurvey only”, because no isolate is reported for those detections. Where sites coincide at this scale, markers are displaced by up to about two degrees so that each remains distinguishable. The concentration of detections in Southeast Asia reflects the distribution of surveillance and diagnostic capacity as well as that of the virus; absence of a marker indicates absence of published detection, not established absence of PRV. Compiled from Table 3; base map from Natural Earth.

Genomic analysis of this cluster placed the causative strains within the recognized diversity of PRV and extended the documented range of disease into the Indian subcontinent, the first reported association of PRVs with human encephalitis; the outbreak is described in detail in Section 3.3 [38]. These findings, together with the increasing global detection of PRVs, are closely linked to accelerating environmental and ecological changes that enhance opportunities for zoonotic spillover [48]. However, the true epidemiological burden of PRVs remains uncertain because most available data derive from isolated surveillance studies, outbreak investigations, or travel-associated cases, introducing substantial geographic and sampling bias [36]. The apparent concentration of reported cases in Southeast Asia may therefore partly reflect differences in diagnostic capacity and surveillance intensity rather than restricted viral circulation [36]. In addition, the absence of routine PRV testing in respiratory diagnostic panels likely contributes to significant underrecognition of mild or asymptomatic infections, limiting accurate assessment of transmission dynamics and spillover frequency [49]. Anthropogenic factors such as deforestation, climate-driven habitat shifts, urbanization, and habitat fragmentation are also reshaping bat ecology and increasing human exposure to reservoir hosts [50]. These ecological disruptions may promote synchronized viral shedding within bat populations and facilitate the establishment of large urban bat colonies, thereby increasing the risk of recurrent zoonotic transmission events [51]. The global distribution of documented PRV detections and the chronology of their reporting are summarized in Figure 4.

### Summary of documented human PRV outbreaks and serological studies

To facilitate comparison of the global epidemiological record, Table 3 consolidates available data on documented human PRV infections and major serological studies. The table highlights the fragmented nature of current surveillance: case fatality rates are rarely systematically reported, seroprevalence estimates are available only for selected populations, and transmission routes remain presumed rather than confirmed in most clusters. Where seroprevalence data do exist, methodological heterogeneity limits comparability: the Singapore and Malaysia occupational serosurvey relied on plaque-reduction neutralization titres with cohort-specific cutoffs and a modest sample size, precluding population-level extrapolation [37]; cross-reactivity among PRV strains and, potentially, with other fusogenic orthoreoviruses has not been systematically excluded in most assays; and none of the available serosurveys report assay sensitivity or specificity against a validated reference standard [49], so reported seropositivity should be interpreted as an approximate rather than precise estimate of true exposure. Where data are lacking, this absence itself constitutes an important finding that should motivate prospective cohort studies and standardized outbreak reporting protocols. The distinction between endemic circulation (sustained, recurring detection within a defined population or region) and epidemic events (a clear increase above baseline, often with a defined point source) is important for interpreting these data. Current evidence suggests PRVs are endemically circulating in bat populations across the Old World, with episodic human spillover events rather than sustained human-to-human transmission chains.

A particularly significant recent development concerns the Bangladesh cluster of 2022–2023, which constitutes the most clinically severe PRV outbreak reported to date and warrants detailed discussion. The cluster, described by Islam et al. in Emerging Infectious Diseases (2025), involved at least 11 cases of acute respiratory disease and encephalitis in three districts of Bangladesh, with a reported case fatality rate of 36%, substantially higher than in previous Malaysian clusters, where fatalities were not documented [38]. Genomic characterization identified bat reovirus strains with 96% nucleotide identity to the Indonesian 2010 isolate, consistent with *Pteropine orthoreovirus* [38]. Palm sap harvesting was identified as the primary suspected transmission route, representing the first documented bat-to-human spillover via a food vehicle for PRVs [52]. This pathway, analogous to the Nipah virus transmission route in Bangladesh, underscores the importance of occupational exposure and food-safety interventions at the human-wildlife interface. The Bangladesh cluster remains the first association of PRV with fatal human encephalitis, fundamentally expanding the recognized clinical spectrum beyond respiratory disease. It also raises unresolved questions regarding whether the higher case fatality reflects strain-specific virulence, differences in host immune status, delayed diagnosis, or limited intensive-care capacity in the affected settings, all areas requiring urgent investigation. Clinically, the Bangladesh cases presented with fever, altered consciousness, and seizures progressing to encephalitis, in several cases following a prodromal respiratory illness, and were fatal in over one-third of patients; this contrasts with the Malaysian Kampar and Melaka clusters, in which patients presented with influenza-like illness and acute respiratory tract infection without documented encephalopathy or fatality. The reasons for this divergent clinical spectrum are not established and may reflect strain-specific neurotropism, differences in host susceptibility or co-morbidity, or ascertainment bias toward more severe presentations in a setting with limited routine PRV testing; distinguishing between these possibilities is a priority for future comparative pathogenesis studies.

### One health control measures at the human-animal-ecosystem interface

Translating the One Health framework from a conceptual model into actionable PRV control requires interventions operating simultaneously at three levels: the ecosystem-wildlife interface, the production system and human-animal interface, and the community public health level. At the ecosystem and wildlife level, the primary intervention is active bat colony surveillance integrated with habitat monitoring. Longitudinal studies tracking PRV shedding prevalence and intensity within pteropodid colonies across seasons would allow identification of high-risk periods, particularly reproductive seasons associated with synchronized shedding, and inform timing of risk-reduction measures. Habitat conservation policies that limit encroachment into bat roosting areas and prevent forest fragmentation represent upstream interventions with the potential to reduce spillover opportunities at their source [50,51]. Ectoparasite surveillance, particularly of bat flies (*Eucampsipoda sundaica*) that have been shown to harbour PRV, should be incorporated into wildlife monitoring protocols, as arthropod vectors may contribute to intra-colony viral maintenance and potentially to bridging transmission [40]. The evidence base for evaluating these measures remains limited; systematic ecological studies are needed to determine which habitat interventions most effectively reduce spillover risk.

At the production system and human-animal interface, the Bangladesh 2022–2023 cluster highlights palm sap harvesting as a high-priority intervention target. In Bangladesh, date palm sap is traditionally collected in open containers overnight, allowing bat excreta to contaminate the sap, a route demonstrated for Nipah virus and now implicated for PRV [52]. Covering palm sap collection pots with bamboo skirts, a low-cost measure already promoted in Nipah prevention campaigns, represents an evidence-based, immediately implementable intervention that should be evaluated and extended to PRV prevention efforts. Market controls in areas of frequent bat-human contact and inspection of wildlife trade chains are additional interface-level measures warranted by the ecological data, though their efficacy for PRV specifically has not been formally evaluated. In Malaysia, the recurrent identification of PRV in outpatient settings in Kuala Lumpur and other urban centres [36] suggests that healthcare workers managing patients with unexplained respiratory or neurological illness in PRV-endemic areas should be aware of the diagnosis and apply standard droplet precautions during the initial evaluation period when the etiology is unknown.

At the community and public health level, risk communication and community engagement are essential complements to technical interventions. Education campaigns targeting populations at highest risk of bat contact, including agricultural workers, forest-adjacent communities, and cave-visiting tourists, can reduce exposure through behavioural modifications such as avoiding handling of bats, covering food and water sources in bat-frequented areas, and seeking care promptly when respiratory or neurological symptoms follow a potential exposure. Contact tracing protocols implemented during the Kampar outbreak in 2006 demonstrated feasibility and identified limited secondary transmission [33], providing a model that should be formalized into national outbreak response plans in Malaysia, Indonesia, Bangladesh, and other countries in the PRV endemic region. Quantitative estimates of community-level intervention effectiveness remain largely unavailable; the establishment of prospective surveillance cohorts in high-risk communities would allow rigorous evaluation of both exposure-reduction measures and healthcare-seeking behaviours. Every control measure described above nonetheless depends on the ability to recognize infection in the first place, which returns the discussion to diagnosis and clinical management.

## Diagnostic approaches, therapeutic development, and novel applications

**Figure 5.**
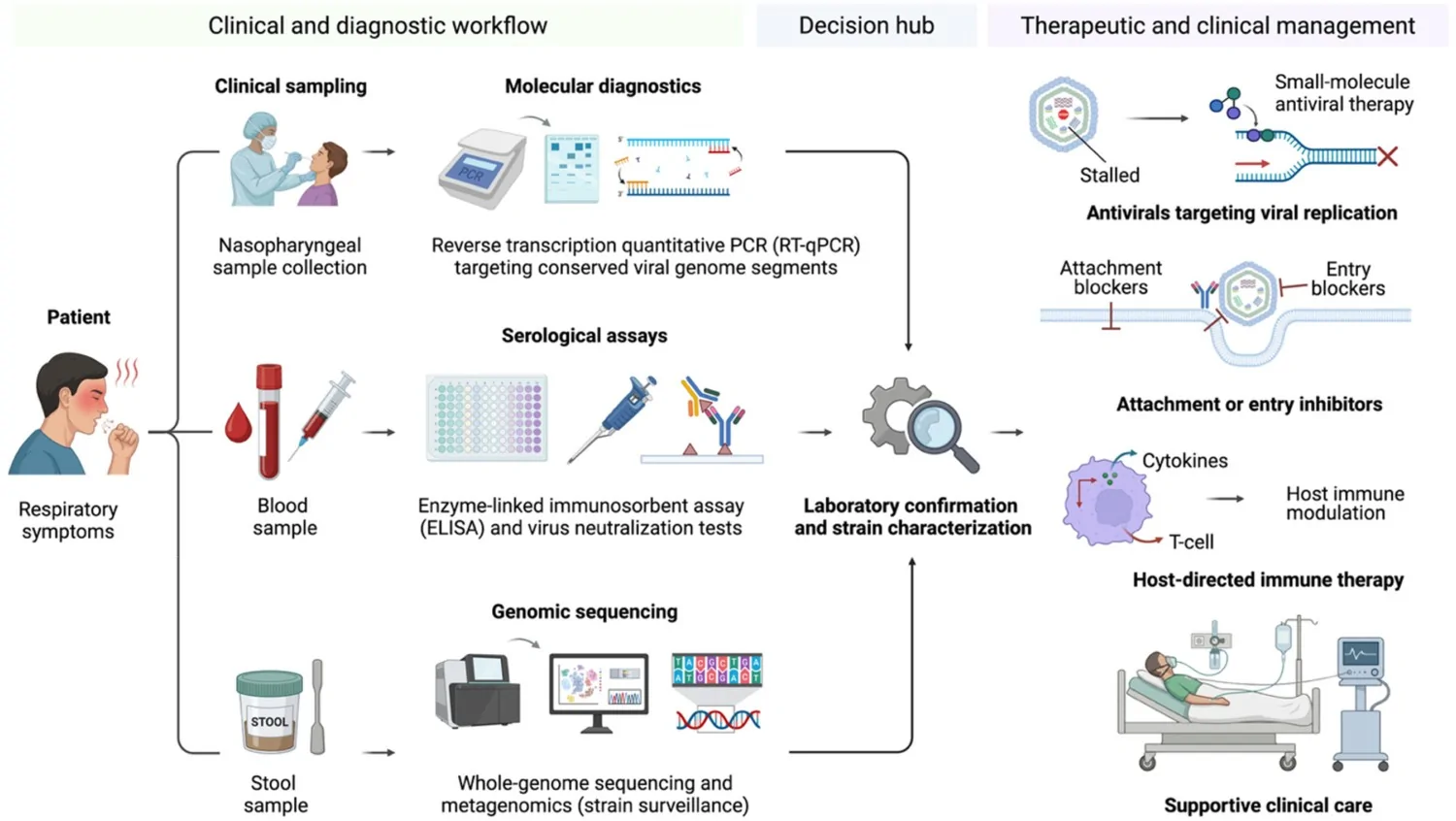
Clinical diagnostic workflow and therapeutic management of *Pteropine orthoreovirus* (PRV) infection. The algorithm outlines a multi-tiered diagnostic and management approach for suspected PRV infection. Patients presenting with acute respiratory illness or unexplained encephalitis, particularly with relevant exposure history, undergo specimen collection for molecular testing. Real time quantitative polymerase chain reaction targeting conserved PRV genomic regions enables rapid diagnosis, while serological assays and virus isolation provide confirmatory evidence when needed. Confirmed cases receive supportive care, and investigational therapies, including repurposed antivirals and host-targeted agents, are highlighted as potential treatment strategies. The surveillance context in which these assays are deployed is shown in Figure 3. Created in BioRender. Ahmed, M. M. (2026) https://BioRender.com/p5myfpx.

Because PRV infection is clinically indistinguishable from many acute respiratory tract infections, diagnosis relies on a tiered and integrated framework that combines epidemiological suspicion with laboratory confirmation [10]. In clinical practice, suspected cases are initially evaluated using nucleic acid-based assays, primarily real-time quantitative polymerase chain reaction (RT-qPCR) on respiratory specimens and, when indicated, additional sample types, complemented by serology for retrospective exposure assessment and viral culture when definitive characterization is required [53]. At the laboratory level, RT-qPCR assays typically target conserved genomic regions, often within S-class segments encoding key virulence and host-interaction determinants and represent the most practical approach for acute case confirmation [54]. Given the segmented nature of the PRV genome and the high frequency of reassortment, sequencing approaches, either targeted or whole genome, are particularly valuable for strain identification, molecular epidemiology, and outbreak investigation when resources permit [10]. Serological methods, including ELISA with confirmatory neutralization testing, support retrospective diagnosis and population-level exposure estimates [49]. For definitive virological confirmation and phenotypic characterization, virus isolation in permissive cell lines remains an option, with PRVs often producing a characteristic fusogenic cytopathic effect [55]. An overview of a practical diagnostic workflow and current management options is summarized in Figure 5. Published PRV-specific RT-qPCR and serological assays generally report analytical sensitivity in the range of low copy numbers per reaction under laboratory conditions; however, head-to-head clinical sensitivity and specificity data against a confirmed-case reference standard are not available in the current literature.

Despite growing recognition of PRVs as emerging zoonotic pathogens, clinical management remains largely supportive, as no PRV-specific antivirals or licensed human vaccines are currently available [52]. Nevertheless, therapeutic innovation has progressed through drug-repurposing strategies and host-target discovery approaches. Several candidate inhibitors with in vitro activity have been identified, including compounds that interfere with viral replication or release and agents that constrain host nucleotide biosynthesis [28]. The identification of HS as a key cellular attachment factor supports the concept of entry inhibition, such as the use of HS competitors, as a potential early-intervention strategy [56]. In parallel, genome-wide genetic screens have highlighted host metabolic dependencies, including folate and one-carbon metabolism pathways, as potentially druggable nodes for antiviral intervention [57]. Although these findings collectively support a pipeline of candidate therapeutics, translation into clinical practice will require standardized experimental models, rigorous pharmacological characterization, and comprehensive safety evaluation. A major barrier to PRV-specific therapeutic development is the absence of a standardized, internationally validated animal model with a clinical endpoint matching human disease, spanning both the respiratory and, following the Bangladesh cluster, encephalitic presentations; the BALB/c respiratory model does not reproduce neurological involvement, and the lack of harmonized challenge doses and endpoints across laboratories complicates comparison of candidate antivirals.

Experimental systems for PRV research remain limited but are expanding. In vitro, PRV strains replicate efficiently in Vero and Vero E6 cells, which remain the standard platform for isolation and titration, while replication in bat-derived cell lines is strain-dependent and generally less cytopathic, consistent with the asymptomatic reservoir phenotype [55]. In vivo, the BALB/c mouse model, established by intranasal inoculation with human- and bat-derived strains, reproduces acute respiratory disease and has been used to demonstrate protective immunity following non-lethal challenge [20,58]. More recently, reverse-genetics-derived recombinant viruses in BALB/c mice have been used to map virulence determinants to the S1 and S2 segments, with σA-dependent lung pathology and cytokine dysregulation observed following intranasal challenge [29]. Cynomolgus macaques have also been experimentally infected and seroconvert following PRV challenge, supporting their relevance as a non-human primate model bridging rodent studies and human disease [37], and bat-derived intestinal and lung organoids have recently been developed to model tissue-specific replication differences between the reservoir and incidental hosts [59,60]. Each model has limitations: Vero cell culture does not capture innate immune responses relevant to pathogenesis; the BALB/c model, while tractable, may not fully recapitulate the encephalitic presentation reported in the Bangladesh cluster; and macaque and organoid models remain resource-intensive and are not yet standardized across laboratories.

Vaccine development, while still at a preclinical stage, is supported by compelling experimental evidence demonstrating that protective immunity against PRVs is achievable [20]. In murine models, intranasal inoculation with a non-lethal PRV dose confers complete protection against subsequent lethal challenge, establishing proof of principle for vaccine-induced immunity [20]. Current investigational strategies focus on DNA vaccines encoding immunogenic viral proteins, such as the outer capsid attachment protein σC and the non-structural protein σNS [61]. However, the high genetic variability of σC poses a significant challenge for universal vaccine design, driving interest in innovative antigen production and presentation platforms, including wheat germ cell-free systems that promote proper protein folding and enhance the induction of neutralizing antibodies [61,62]. Passive immunotherapy remains an unexplored avenue for PRV, although active immunization has been shown to confer complete protection against lethal challenge in murine models [20].

Beyond their role as pathogens, PRVs have attracted considerable attention as potential oncolytic agents due to their intrinsic ability to selectively infect and lyse cancer cells while sparing normal tissues [35]. This oncolytic tropism is partly attributed to the frequent dysregulation of the Ras signalling pathway in malignant cells, which suppresses innate antiviral responses and facilitates viral replication [35]. The Sikamat virus (PRV7S), a Malaysian isolate, has been extensively characterized as a potent oncolytic virus against nasopharyngeal carcinoma (NPC) [35]. Evidence demonstrates that PRV7S exhibits efficacy comparable to or exceeding that of mammalian orthoreovirus type 3, inducing profound growth inhibition and apoptosis, reaching up to 98% in certain NPC cell lines, while exerting minimal cytotoxic effects on non-cancerous nasopharyngeal cells [35]. Molecular analyses reveal that PRV7S infection leads to downregulation of Ras proteins and activation of executioner caspases such as caspase-3, consistent with programmed cell death pathways [35].

A notable advantage of bat-derived orthoreoviruses like PRV7S in oncolytic virotherapy is the negligible pre-existing immunity in human populations, allowing for systemic administration without rapid immune clearance and potentially improved delivery to metastatic sites [35]. Moreover, the potent FAST-mediated syncytium formation characteristic of PRVs may amplify oncolytic efficacy through bystander killing of neighbouring malignant cells [13]. Parallel advances in molecular virology have transformed PRVs into versatile biotechnological platforms and research tools [16]. The development of plasmid-based reverse genetics systems has enabled the generation of recombinant PRVs entirely from cloned cDNA, allowing precise manipulation of the viral genome [63]. This technology facilitates the creation of reporter viruses expressing markers such as green fluorescent protein (GFP) or luciferase for high-throughput antiviral screening and real-time in vivo tracking of infection and it also opens avenues for engineering PRVs as vaccine vectors or targeted gene delivery systems [16].

Additionally, the unique fusogenic properties of PRV FAST proteins are being explored for broader biotechnological applications, including the development of fusogenic liposomes for enhanced drug delivery and tissue engineering strategies aimed at promoting controlled cell fusion [11]. To better model the complex dynamics of zoonotic spillover and host specificity, advanced ex vivo systems based on bat-derived organoids have been developed. Bat intestinal organoids and lung organoids provide physiologically relevant platforms to investigate viral tropism, replication kinetics, and host responses in natural reservoir tissues [59,60]. These models demonstrate marked differences in PRV replication, with efficient replication in bat intestinal tissues but limited replication in lung tissues, reflecting asymptomatic infection in bats and severe respiratory disease in humans [59,60]. Such findings provide important insight into cross-species transmission and pathogenicity.

Overall, the expanding understanding of PRVs highlights their significance as both emerging zoonotic threats and potential biomedical tools. Their geographic spread, genomic diversity, and evolving clinical spectrum emphasize the need for integrated One Health surveillance, improved molecular diagnostics, and ecological monitoring. At the same time, advances in antiviral strategies and oncolytic applications illustrate the broader translational potential of PRV research for human health.

## Conclusions

This review has synthesized current knowledge on PRV molecular pathogenesis, global epidemiology, and One Health control dimensions, revealing both what is known and where critical gaps persist. Among the priorities identified, three are particularly actionable and time-sensitive. First, the Bangladesh 2022–2023 cluster (Section 3.3) demands urgent follow-up: prospective cohort studies are needed to determine whether this high fatality reflects strain-specific virulence or contextual factors such as delayed diagnosis and limited intensive-care access, and targeted palm sap safety campaigns modelled on existing Nipah interventions should be implemented in affected districts. Second, the systematic underdetection of PRV in routine diagnostic panels is a correctable gap: adding PRV-specific RT-qPCR to sentinel severe acute respiratory infection and encephalitis-of-unknown-etiology surveillance networks in Southeast Asia and Bangladesh would substantially improve epidemiological visibility without requiring new infrastructure. Third, quantitative seroprevalence data from at-risk occupational groups, i.e. bat hunters, cave explorers, fruit farmers, and palm sap tappers, are almost entirely lacking outside Malaysia and Singapore; systematic serosurveys in these populations would allow evidence-based stratification of intervention priorities and genuine estimation of the human burden of PRV. At the therapeutic frontier, the FAST protein fusion mechanism and heparan sulphate-mediated entry represent well-characterized molecular targets for which candidate inhibitors exist in vitro, but none have progressed to standardized animal efficacy studies; establishing internationally agreed-upon animal models and antiviral assay benchmarks should be a near-term priority. Overall, the evidence calls for PRV to be recognized not only as a regional zoonosis but as a globally relevant emerging pathogen, warranting coordinated surveillance, standardized outbreak reporting, and accelerated One Health research at the human-animal-environment interface.

## Data Availability

Not applicable. No new data were analyzed or synthesized in this study.
